# Population-Based Survival Analysis of Patients With Limb Rhabdomyosarcoma and Metastasis at Diagnosis

**DOI:** 10.3389/fsurg.2021.738771

**Published:** 2021-11-04

**Authors:** Chunying Yang, Haiqing Wang, Feng Niu, Lufeng Yao

**Affiliations:** ^1^Department of Neurology, The Affiliated Hospital of Medical School of Ningbo University, Ningbo, China; ^2^Department of Orthopedic Surgery, Ningbo No. 6 Hospital, Ningbo, China; ^3^Department of Orthopedic Surgery, Ningbo Hospital of Traditional Chinese Medicine Affiliated to Zhejiang Chinese Medical University, Ningbo, China

**Keywords:** limb rhabdomyosarcoma (LRMS), metastasis at diagnosis, survival analysis, tumor subtype, radiotherapy

## Abstract

**Purpose:** Given the poor prognosis and the relative rarity of patients diagnosed with limb rhabdomyosarcoma (LRMS) and metastasis at diagnosis, we performed this study to reveal distinctive clinical features and evaluated prognostic factors of this special population in order to provide appropriate treatment.

**Patients and Methods:** We carried out retrospective research of patients diagnosed with LRMS and metastasis from 1975 to 2016 using the Surveillance, Epidemiology, and End Results (SEER) program database. Survival curves were generated by applying the Kaplan–Meier method. In terms of evaluating and determining independent predictors of survival, we conducted univariate and multivariate survival analyses using the Cox proportional hazard regression model.

**Results:** This retrospective analysis contained a series of 245 patients with metastatic LRMS, with male predominance (male vs. female, 1.6:1). Nearly half of the patients were diagnosed with alveolar rhabdomyosarcoma (44.9%). According to the results of the univariate and multivariate analyses, younger age, tumor subtype, and radiotherapy were found to be significantly associated with improved overall survival (OS) and cause-specific survival (CSS).

**Conclusions:** Patients with LRMS and metastasis at diagnosis experienced a quite poor prognosis. Age at diagnosis, tumor subtype, and radiotherapy can help clinicians to better estimate the prognosis. This study indicated that local radiotherapy can provide a survival benefit.

## Introduction

Rhabdomyosarcoma (RMS) is the most common soft tissue sarcoma during childhood (1), and derives from the mesenchymal tissue. A limb is one of the most commonly affected sites of RMS, accounting for 18% (2). The prognosis of limb RMS (LRMS) was poorer than that of RMS at other sites (3–6). Tumor size, site, staging, age of patient, nodal involvement, and alveolar subtype (ARMS) were strongly associated with the prognosis of patients with RMS (5, 7–9). Distant metastasis is a difficult and common problem for patients with RMS, with a rate ranging from 14 to 28% (10, 11). Bone and lung are the most common organs of metastasis (12). Kim et al. (12) reported that most patients with metastatic RMS had metastatic lesions identified at the time of initial presentation. Additionally, they confirmed that age at diagnosis and site of the primary tumor were significant independent predictors of distant metastasis.

Current multidisciplinary treatments for RMS include surgical resection, chemotherapy, and radiotherapy. Although multimodal treatments were performed in patients with RMS, the prognosis for patients who developed metastasis is still far worse (13). LRMS with metastasis at presentation is an unusual situation. To our knowledge, prognostic predictors of this population are poorly understood because of their rarity. Therefore, we performed this study to identify useful prognostic predictors and explore the appropriate treatment to improve the survival of patients with LRMS.

## Materials and Methods

### Patient Population

This analysis included 245 patients diagnosed with LRMS and metastasis at from 1975 to 2016. We used the case-listing procedure to obtain all patients information from the public Surveillance, Epidemiology, and End Results (SEER) database. This study was in accordance with standard rules and approved by our hospital ethics committee. We selected patients with LRMS following International Classification of Diseases for Oncology, 3rd edition (ICD-O-3): morphology code, 8900–8902, 8910, 8912, 8920, and primary site code, C49.1-C49.2. The inclusion criteria were as follows: (1) RMS diagnosis based on histology; (2) patients with distant diseases. The exclusion criteria were as follows: (1) RMS diagnosis only based on clinical manifestations, imaging findings, autopsy, or death records; (2) Patients with missing survival information. Patient data included age, sex, histological type, tumor size, surgery, radiotherapy, chemotherapy, vital status, death cause, and survival month. In the current study, all the patients had metastasis at the initial diagnosis and were in the M1 stage. After diagnosis, they received their treatments. Surgery or radiotherapy for metastatic LRMS in our research refers to management for primary lesions.

### Statistical Analyses

All statistical analyses were performed using the SPSS version 21.0 software (IBM, Chicago, IL, United States). Cause-specific survival (CSS) was regarded as the date from diagnosis to death due specifically to RMS (14). Survival curves, namely, overall survival (OS) and CSS, were constructed using the Kaplan–Meier method. In order to evaluate and determine the independent predictors of survival, we performed univariate and multivariate analyses using the Cox proportional hazard regression model. We used the “enter” parameter during the Cox regression analysis. We also calculated hazard ratios (HRs) with 95% confidence intervals (CIs) of each predictor to present their influences on survival. There were only six patients with spindle cell RMS (2.4%)and one patient with mixed-type RMS (0.4%) (Table 1). In order to rationally perform univariate and multivariate analyses, we combined these two types of RMS and alveolar RMS into “other type” for further survival analysis. In our analyses, statistical significance was achieved if the bilateral *P*-value was <0.05.

**Table 1:** Baseline characteristics of 245 patients with limb rhabdomyosarcoma and metastasis at diagnosis.

**Variable**	**Value**
**Age (years)**	
<20	134 (54.7%)
20–40	39 (15.9%)
>40	72 (29.4%)
**Sex**	
Female	95 (38.8%)
Male	150 (61.2%)
**Tumor site**	
Upperlimb	79 (32.2%)
Lower limb	166 (67.8%)
**Tumor type**	
Rhabdomyosarcoma, NOS	49 (20.0%)
Pleomorphic rhabdomyosarcoma, adult type	45 (18.4%)
Embryonal rhabdomyosarcoma, NOS	34 (13.9%)
Alveolar rhabdomyosarcoma	110 (44.9%)
Spindle cell rhabdomyosarcoma	6 (2.4%)
Mixed type rhabdomyosarcoma	1 (0.4%)
**Tumor size**	
<5 cm	30 (12.2%)
5–10 cm	80 (32.7%)
>10 cm	65 (26.5%)
Unknown	70 (28.6%)
**Surgery**	
Yes	106 (43.3%)
No	139 (56.7%)
**Radiotherapy**	
Yes	118 (48.2%)
No	127 (51.8%)
**Chemotherapy**	
Yes	205 (83.7%)
No	40 (16.3%)
**Dead**	
Yes	200 (81.6%)
No	45 (18.4%)
**3-year OS rate**	21.0%
**3-year CSS rate**	23.0%
**5-year OS rate**	10.9%
**5-year CSS rate**	14.1%

*OS, overall survival; CSS, cancer-specific survival*.

## Results

### Baseline Characteristics

This retrospective analysis included a total of 245 patients with metastatic LRMS derived from the SEER database. Basic clinical and pathological features of patients with metastatic LRMS are shown in Table 1. According to age at diagnosis, patients were divided into three groups: <20 years, between 20 and 40 years, and over 40 years. Over half of the patients were aged <20 years. The population consisted of 95 (38.8%) females and 150 (61.2%) males. Tumor location distribution was upper limb 32.2% and lower limb 67.8%. Nearly half of the patients were diagnosed with alveolar rhabdomyosarcoma (44.9%). We divided the tumor size into four subtypes: <5 cm (12.2%), 5–10 cm (32.7%),>10 cm (26.5%), and unknown (28.6%). Less than half of the patients (43.3%) had local surgery and radiotherapy (48.2%), and the majority of the patients (83.7%) received chemotherapy. This population had worse survival with a 5-year OS and CSS rate of 10.9 and 14.1%, respectively.

### Univariate Survival Analysis

**Figure 1:**
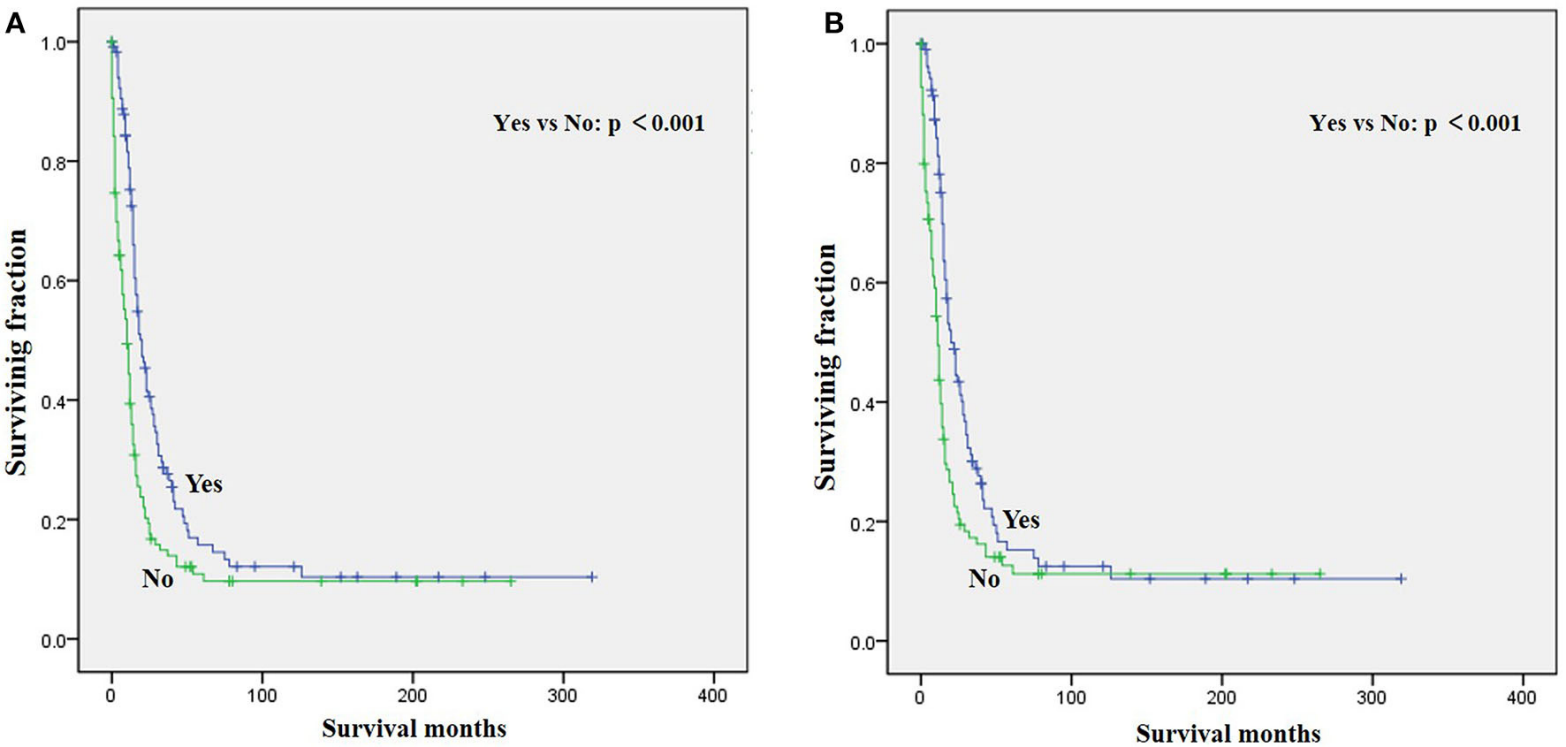
Graphs show Kaplan–Meier curves of **(A)** overall survival (OS) and **(B)** cause-specific survival (CSS) in patients with limb rhabdomyosarcoma (LRMS) and metastasis at diagnosis stratified by radiotherapy.

**Figure 2:**
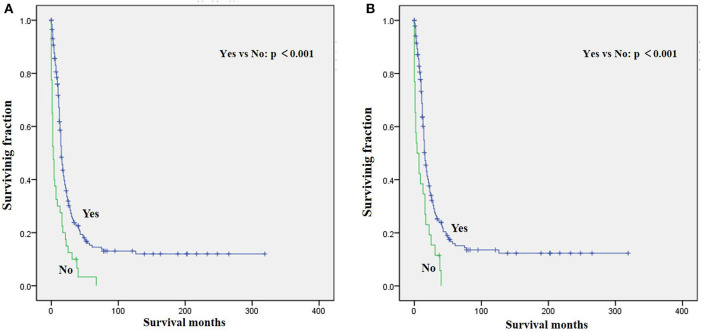
Graphs show Kaplan–Meier curves of **(A)** OS and **(B)** CSS in patients with LRMS and metastasis at diagnosis stratified by chemotherapy.

**Figure 3:**
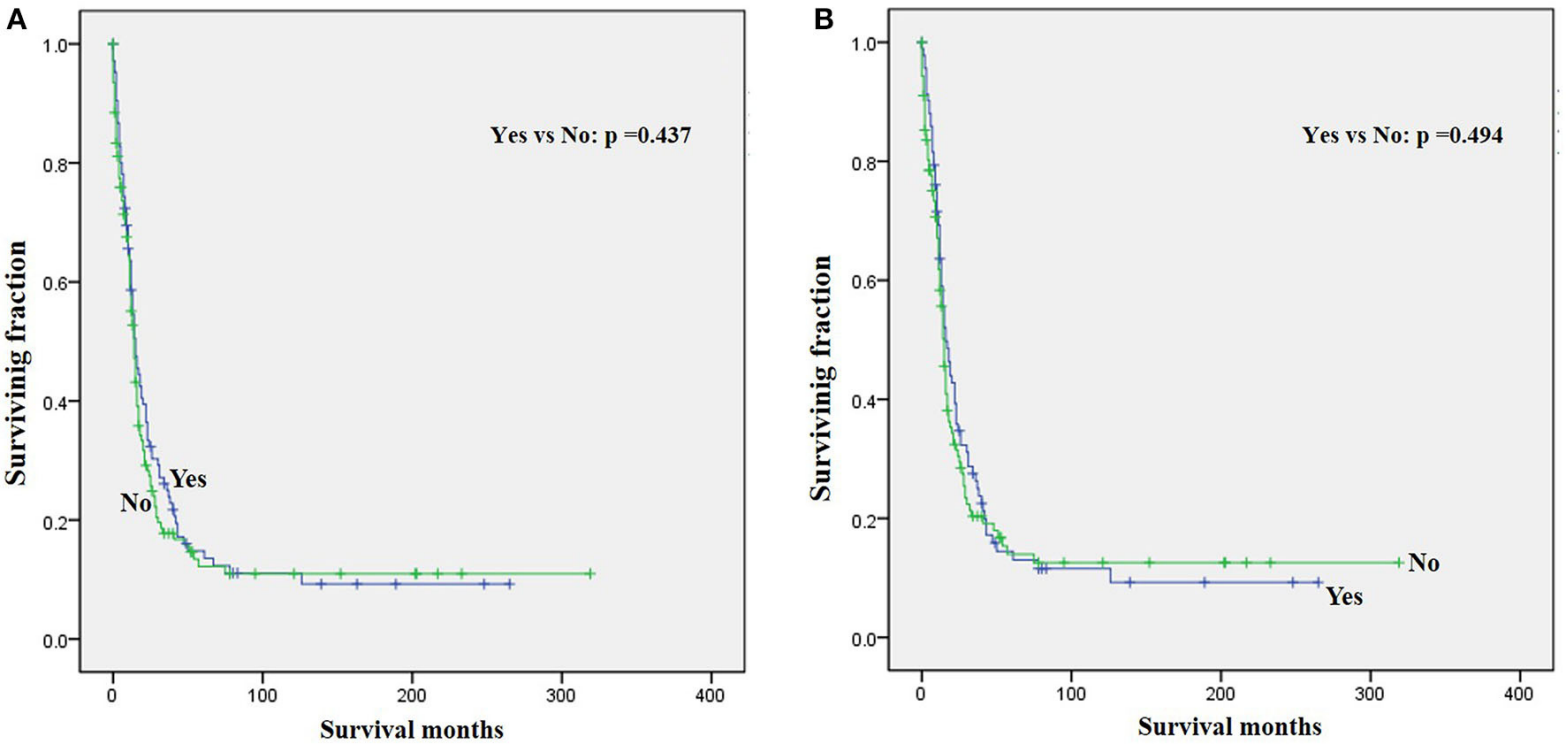
Graphs show Kaplan–Meier curves of **(A)** OS and **(B)** CSS in patients with LRMS and metastasis at diagnosis stratified by surgery.

The univariate analysis (Table 2) revealed that gender, tumor location, and tumor size were not significant predictors of either OS or CSS. Younger age was significantly predictive of improved survival. Tumor type was a meaningful predictor of OS and CSS, with embryonal rhabdomyosarcoma, NOS, and other types predicting a better prognosis. Patients who received radiotherapy or chemotherapy experienced significantly better outcomes than the other patients (Figures 1, 2). However, local surgery did not significantly increase OS or CSS of the patients (Figure 3).

**Table 2:** Univariate Cox analysis of variables in patients with limb rhabdomyosarcoma and metastasis at diagnosis.

**Variable**	**OS**		**CSS**	
	**HR (95% CI)**	***P***	**HR (95% CI)**	***P***
**Age (years)**				
<20	1		‘	
20–40	2.107 (1.428–3.109)	<0.001	2.098 (1.407–3.129)	<0.001
>40	3.252 (2.357–4.487)	<0.001	3.132 (2.167–4.528)	<0.001
**Sex**				
Female	1		1	
Male	1.122 (0.843–1.495)	0.430	1.095 (0.804–1.491)	0.565
**Tumor site**				
Upperlimb	1		1	
Lower limb	0.917 (0.683–1.231)	0.566	0.884 (0.646–1.209)	0.440
**Tumor type**				
Rhabdomyosarcoma, NOS	1		1	
Pleomorphic rhabdomyosarcoma, adult type	1.405 (0.775–1.751)	0.116	1.708 (1.052–2.772)	0.030
Embryonal rhabdomyosarcoma, NOS	0.458 (0.579–1.756)	0.002	0.498 (0.293–0.846)	0.010
Other types	0.420 (0.289–0.610)	<0.001	0.460 (0.307–0.691)	<0.001
**Tumor size**				
<5 cm	1		1	
5–10 cm	1.103 (0.690–1.761)	0.683	1.116 (0.677–1.840)	0.666
>10 cm	1.273 (0.779–2.079)	0.336	1.264 (0.747–2.141)	0.383
**Surgery**				
Yes	1		1	
No	1.115 (0.843–1.474)	0.446	1.109 (0.821–1.498)	0.502
**Radiotherapy**				
Yes	1		1	
No	1.772 (1.340–2.345)	<0.001	1.679 (1.242–2.269)	0.001
**Chemotherapy**				
Yes	1		1	
No	2.387 (1.677–3.399)	<0.001	2.176 (1.418–3.340)	<0.001

### Multivariate Survival Analysis

Variables with *p* <0.1 from the univariate analysis were subsequently integrated into the multivariable Cox analyses. The multivariate analysis (Table 3) showed that age, tumor type, and radiotherapy were identified as meaningful independent predictive variables of both OS and CSS.

**Table 3:** Multivariate Cox analysis for OS and CSS of patients with limb rhabdomyosarcoma and metastasis at diagnosis.

**Variable**	**OS**		**CSS**	
	**HR (95% CI)**	***P***	**HR (95% CI)**	***P***
**Age (years)**				
<20	1		1	
20–40	1.846 (1.225–2.782)	0.003	1.803 (1.177–2.762)	0.007
>40	1.779 (1.130–2.801)	0.013	1.755 (1.061–2.903)	0.029
**Tumor type**				
Rhabdomyosarcoma, NOS	1		1	
Pleomorphic rhabdomyosarcoma, adult type	1.040 (0.665–1.625)	0.865	1.268 (0.774–2.138)	0.331
Embryonal rhabdomyosarcoma, NOS	0.461 (0.272–0.780)	0.004	0.486 (0.280–0.843)	0.010
Other types	0.507 (0.335–0.767)	0.001	0.530 (0.340–0.827)	0.005
**Radiotherapy**				
Yes	1		1	
No	1.803 (1.348–2.411)	<0.001	1.670 (1.222–2.281)	0.001
**Chemotherapy**				
Yes	1		1	
No	1.194 (0.774–1.841)	0.424	1.022 (0.610–1.714)	0.933

## Discussion

Rhabdomyosarcoma is a complex soft tissue malignancy with childhood propensity. At present, although over 70% of local RMS is cured (15), the treatment of metastatic patients is still a thorny problem, with the overall cure rate being <30% (16). Due to the relative rarity and neglect of metastatic RMS, few studies reported their clinical characteristics and survival outcomes. Patients with metastatic RMS usually had significantly poorer clinical outcomes (17). This study also revealed that the survival results of metastatic LRMS are considered unfavorable. In order to improve the survival of patients with metastatic LRMS, we conducted this study and analyzed a series of 245 cases to identify the survival predictors using the SEER database.

Based on the results of the univariate and multivariate analyses, younger age significantly predicted an improved survival among patients with metastatic LRMS, which was in agreement with some previous studies (18–20). Additionally, the multivariate analysis revealed that older age at diagnosis was also a positive independent predictor of distant metastasis (12). Maybe the pathogenesis, biological behavior, and therapeutic effect of patients with different age groups are also different (21). However, Bergamaschi et al. (22) demonstrated that age was not associated with survival among adult RMS based on univariate analysis. In our series, the univariate analysis showed no significant correlation between sex and survival, which was supported by several studies (20, 22). Additionally, male predominance was found for metastatic LRMS (male vs. female, 1.6:1). Similarly, we found that the tumor site did not affect survival significantly.

Tumor size has been demonstrated as an important prognostic factor in the previous literature (22, 23). However, this study demonstrated that tumor size was not associated with survival. Maybe patients with metastatic RMS have distinctive pathology and genomics compared with patients with localized RMS. Emmanuelle et al. (19) also reported that tumor size was not a risk factor of survival in patients with either localized RMS or advanced RMS. Further research studies are needed to confirm this finding. Some published studies documented the prognostic role of tumor subtype in patients with RMS (19). However, Li et al. (24) hold that tumor subtype was not a survival predictor of sinonasal RMS. Odile et al. (25) did not report a correlation between tumor subtype and survival in localized extremity RMS. In our patients, tumor subtype significantly and independently predicted the survival of patients with metastatic LRMS.

Surgery, radiotherapy, and chemotherapy are still the three cornerstones of treatment for RMS (26). Complete surgical excision is seen as the mainstay of therapy for RMS (1, 23). However, our results demonstrated that surgery for the primary disease was not associated with survival in patients with metastatic LRMS. Radiotherapy was frequently delivered in localized LRMS and approved to be a positive independent survival predictor (23, 26), which also improved survival significantly in those patients with metastatic LRMS. However, radiotherapy might increase metastatic potential in the preclinical model of metastatic behavior of rhabdomyosarcoma xenografts (27). Chemotherapy has been recognized as a popular treatment for advanced and metastatic RMS (28, 29). However, our multivariable analysis indicated that chemotherapy cannot significantly increase the survival of patients with metastatic LRMS. However, it significantly increases median survival, as shown in Figure 2. Prospective, multicenter clinical studies should be performed to further investigate the treatment modalities for patients with metastatic LRMS.

Some limitations should be noted in this study. First, its retrospective nature may cause bias in the prediction of survival. Second, the lack of information about detailed treatment modalities may also affect survival. Third, data on recurrence or metastasis during follow-up are not available in the SEER database. Finally, the database does not provide information on treatment for metastatic sites and recurrence during follow-up.

## Conclusions

This study offers insight into the clinical characteristics and treatment strategies of metastatic LRMS. However, clinical trials are urgently needed to clarify these findings and improve the survival of this special population.

## Data Availability Statement

The raw data supporting the conclusions of this article will be made available by the authors, without undue reservation.

## Ethics Statement

Ethical review and approval was not required for the study on human participants in accordance with the local legislation and institutional requirements. Written informed consent for participation was not required for this study in accordance with the national legislation and the institutional requirements.

## Author Contributions

LY and CY conceived and designed the study. CY, HW, and FN collected the data and performed the statistical analyses. CY wrote the manuscript. LY and HW revised it. All authors have read and approved the final version of the manuscript.

## Funding

This study was performed with the support of the Zhejiang Medical Science & Technology Program (2021KY329 and 2020KY898), Ningbo Yinzhou Science & Technology Program (2020AS0073), and Ningbo Science & Technology Program (Ningbo Natural Science Foundation 202003N4243).

## Conflict of Interest

The authors declare that the research was conducted in the absence of any commercial or financial relationships that could be construed as a potential conflict of interest.

## Publisher's Note

All claims expressed in this article are solely those of the authors and do not necessarily represent those of their affiliated organizations, or those of the publisher, the editors and the reviewers. Any product that may be evaluated in this article, or claim that may be made by its manufacturer, is not guaranteed or endorsed by the publisher.
